# Retrieval of Individual Participant Data for Exercise Meta-Analyses May Not Be Worth the Time and Effort

**DOI:** 10.1155/2016/5059041

**Published:** 2016-03-08

**Authors:** George A. Kelley, Kristi S. Kelley

**Affiliations:** School of Public Health, Department of Biostatistics, West Virginia University, Morgantown, WV 26506-9190, USA

## Abstract

*Purpose*. While individual participant data (IPD) meta-analyses are considered the gold standard for meta-analysis, the feasibility of obtaining IPD may be problematic.* Methods*. Using data from a previous meta-analysis of 29 studies on exercise in adults with arthritis and other rheumatic diseases, the percentage of studies in which useable IPD was provided was calculated.* Results*. Eight of 29 authors (28%, 95% CI = 11% to 44%) provided IPD. Using logistic regression, neither year of publication (odds ratio = 1.05, 95% CI = 0.90 to 1.27, *p* = 0.58) nor country (odds ratio = 1.36, 95% CI = 0.20 to 10.9, *p* = 1.00) was significantly associated with the obtainment of IPD.* Conclusions*. The retrieval of IPD for exercise meta-analyses may not be worth the time and effort. However, further research is needed before any final recommendations can be made.

## 1. Introduction

The prevalence of meta-analyses has increased dramatically over approximately the past 25 years. For example, recent PubMed searches by the first author on December 4, 2015, using the keyword “meta-analysis” found that the number of citations increased from 331 in 1990 to 14,329 in 2014 (Supplementary File 1 in Supplementary Material available online at http://dx.doi.org/10.1155/2016/5059041). The number of meta-analyses focused on exercise has also increased dramatically over the same time period. Using the keywords “exercise AND meta-analysis”, the number of meta-analyses has increased from 7 in 1990 to 472 in 2014 (Supplementary File 2). While aggregate data (AD) meta-analysis, an approach in which summary statistics from eligible studies are pooled, is still the most common type of meta-analysis, individual participant data (IPD) meta-analysis, an approach that pools the results from different studies based on data from each participant, is considered the gold standard [1–4]. However, in addition to cost [5], a major disadvantage of an IPD meta-analysis may be the ability to retrieve IPD from all eligible studies [6–8]. As a result, this negates many of the potential advantages of an IPD meta-analysis (investigating heterogeneity at the participant level, avoiding ecological bias, and harmonizing different data sources). A previous study of 142 IPD meta-analyses across a variety of different subject areas reported that the obtainment of IPD ranged from 25% to 100% [9]. With respect to exercise, the authors are only aware of one previous study regarding the obtainment of IPD. In this study, the current investigative team reported the retrieval of IPD from only 29 of 76 (38.2%) eligible studies dealing with the effects of exercise on bone mineral density in adults [10]. However, this study was conducted approximately 13 years ago. Since that time, technological advances have improved one's ability to store and share data, thereby making it easier to share deidentified IPD with others. Given that meta-analyses are increasingly used to provide evidence-based decisions regarding the benefits of exercise on a variety of health outcomes, a basic understanding of the best approach for conducting such analyses is needed. Therefore, the purpose of this* brief report *was to contribute to the scant exercise literature by providing up-to-date information regarding the retrieval of IPD, including potential covariates, for a specific meta-analysis.

## 2. Methods

### 2.1. Data Source

Data were derived from a recently published AD exercise meta-analysis in which an IPD meta-analysis was originally planned, details of which have been described elsewhere [11]. Briefly, the specific aims were to calculate a summary effect of exercise on depressive symptoms in adults with arthritis and other rheumatic diseases and explore for potential sources of heterogeneity. Studies were included if they were randomized controlled trials examining the effects of exercise (aerobic, strength training, or both) on depressive symptoms in adults with arthritis and other rheumatic diseases [11]. Twenty-nine studies representing 2,449 participants (1,470 exercise, 979 control) were included [12–40].

### 2.2. Retrieval of IPD

Using a predefined form letter (see Supplementary File 3), deidentified IPD was requested by having the second author contact the corresponding author of each eligible study via electronic mail asking if they would be interested in providing IPD. A response was requested within two weeks, regardless of interest, with the authors being informed that they would be listed in the acknowledgements section of each published study derived from the project if they provided their IPD. If no response was received within two weeks, up to two additional requests were sent via electronic mail. For those who responded but chose not to contribute, reasons given for not participating, if any, were recorded. For those authors who expressed interest in providing IPD, a second electronic mail was sent that included an attachment consisting of a predefined list of IPD requested (see Supplementary File 4). Investigators were asked to provide IPD in a format that was convenient for them within four weeks. If IPD was not received within four weeks, as many as two additional reminders were sent via electronic mail. The dates of all communications were recorded.

### 2.3. Statistical Analysis

Descriptive statistics were used to describe the number of responses to initial electronic mail requests for IPD, number of days to respond to initial electronic mail requests, number of authors who provided useable IPD, and number of days from initial requests to receipt of IPD. Reasons for not providing IPD from authors who were willing to supply such information were also recorded. Furthermore, descriptive statistics were calculated for the two potential predictors included in the regression model.

Because of the small sample size, exact logistic regression was used to examine potential predictors with respect to whether or not IPD was received [41]. Based on the investigative team's previous research [10], the two potential predictors included in the model were country in which the study was conducted and year that the study was published. The chi-square distribution (*χ* ^2^) was used to examine the overall model. The alpha level for statistical significance was set at *p* ≤ 0.05.

## 3. Results

### 3.1. Descriptive Statistics for Retrieval of IPD

The authors from 20 of 29 studies (69.0%, 95% CI = 52.2% to 85.8%) responded to initial electronic mail requests for IPD while 9 (31.0%, 95% CI = 14.2% to 47.8%) never responded despite multiple requests. The response time to initial requests varied widely from 1 to 181 days ($\overset{-}{X}\pm SD$ = 54.2 ± 74.8, 95% CI = 21.4 to 87.0, Mdn = 17). Eight of 29 authors (27.6%, 95% CI = 11.3% to 43.8%) provided IPD. The number of days from initial requests for data to receipt of IPD ranged from 36 to 179 ($\overset{-}{X}\pm SD$ = 74.4 ± 46.4, 95% CI = 42.0 to 106.8, Mdn = 64). Reasons for not providing IPD included no longer having the data (*n* = 4) and time (*n* = 1). Another author said that they would supply IPD if a consortium was formed and in which they were included as a coauthor. Year of publication ranged from 1989 to 2011. Thirteen studies (44.8%, 95% CI = 26.7% to 62.9%) were conducted in the United States while the remaining 16 (55.2%, 95% CI = 37.1% to 73.3%) were conducted in countries other than the United States.

### 3.2. Potential Predictors in the Obtainment of IPD

Results for exact logistic regression are shown in Table 1. The overall model was not statistically significant (*χ* ^2^ = 0.62, *p* = 0.71) and neither year of publication nor country was a significant predictor for the receipt of IPD (*p* > 0.05 for both).

## 4. Discussion

### 4.1. Overall Findings

The current study resulted in a low obtainment of IPD for this exercise meta-analysis, with less than one-third of eligible studies providing IPD. The response rates observed are either similar to [10, 42], lower than, [9, 43] or higher [44] than previous research across a variety of different subject areas. While one might speculate that the formation of a consortium could have increased the response rate, a previous IPD meta-analysis on obesity and diabetes was only able to retrieve approximately 40% of IPD when such a consortium was established [42]. The lack of association between year and country with the obtainment of IPD is in contrast with previous research where a trend was found for both to be associated with the retrieval of IPD [10]. One possible reason for this discrepancy may have been the lack of statistical power because of the smaller number of studies included (29 versus 76) [10]. Finally, and as previously reported in the original meta-analysis [11], no statistically significant or clinically important differences were found in depressive symptoms between those studies that supplied IPD (standardized mean difference (SMD), −0.31, 95% CI, −0.61 to −0.01) and those that did not (SMD, −0.47, 95% CI, −0.65 to −0.28), a finding consistent with the authors' previous research [10].

### 4.2. Implications for Research and Practice

The study reinforces the importance of considering the feasibility of obtaining IPD when deciding whether to conduct an AD or IPD exercise meta-analysis. In addition, the increased costs associated with an IPD meta-analysis need to be considered. For example, Steinberg et al. estimated that the costs associated with conducting a meta-analysis of 12 ovarian cancer studies were more than 5 times greater using the IPD versus AD approach [5]. However, others estimated the costs of this same study to be at least 8 times greater given that the investigative team continued to work on the study after funding for the project ended [45].

### 4.3. Strengths and Potential Limitations

This study provides important up-to-date information regarding the retrieval of IPD for an exercise meta-analysis. This is noteworthy given that this is only the second study that has examined the feasibility of retrieving IPD for an exercise meta-analysis, the first being conducted by the authors back in 2002 [10]. In addition, given that meta-analyses are increasingly used to provide evidence-based decisions regarding the benefits of exercise on a plethora of health outcomes, the results of this study provide critically important information regarding the feasibility of conducting an IPD exercise meta-analysis. However, since the current study focused on one attempted IPD meta-analysis, the findings may not be generalizable to other IPD exercise meta-analyses. In addition, no cost data were collected or analyzed, thereby limiting the applicability of results. Furthermore, the collapsing of countries other than the US into one category because of the small number of studies available for each country could have biased the results. Finally, a better approach for examining potential differences between AD and IPD may have been to include a mixture of AD and IPD rather than dichotomizing the two and comparing them using an AD approach [9, 46]. However, the investigative team was not comfortable using these methods given the inability to obtain the data necessary to replicate the statistical methods and results reported in this previous work (Riley, written communication, July 11, 2012; Staessen, written communication, July 30, 2012) [46].

## 5. Conclusions

The retrieval of IPD for exercise meta-analyses may not be worth the time and effort. However, further research is needed before any final recommendations can be made.

## Supplementary Material

Supplementary File 1: This file provides details regarding a PubMed search conducted on December 4, 2015 for citations indexed between the years 1990 and 2014 using the key term “meta-analysis”.
Supplementary File 2: This file provides details regarding a PubMed search conducted on December 4, 2015 for citations indexed between the years 1990 and 2014 using the key terms “exercise and meta-analysis”.
Supplementary File 3: This file is an example of the initial letter that was sent to authors requesting de-identified individual participant data for their previously published study.
Supplementary File 4: This file is an example of the follow-up letter that was sent to eligible investigators describing what de-identified individual participant data was needed from their previously published study.

## Figures and Tables

**Table 1:** Results for exact logistic regression for receipt of IPD (*n* = 29).

Variable	OR	SE	*p*	95% CI
Year	1.05	0.09	0.58	0.90, 1.27
Country	1.36	1.12	1.00	0.20, 10.90

Note: IPD, individual participant data; OR, odds ratio; SE, standard error; *p*, alpha value, calculated as 2 *∗* the probability of the sufficiency statistic, a statistic derived from single-parameter tests of the null hypothesis that the coefficient equals zero versus a 2-sided alternative; 95% CI, 95% confidence interval; alpha (*p*) and 95% CI calculated from exact conditional distributions; both independent variables (year and country) calculated separately with the other variable conditioned out of the calculation.
