# Supplementary material for: Retrieval of Individual Participant Data for Exercise Meta-Analyses May Not Be Worth the Time and Effort
Source: Biomed Res Int. 2016 Mar 8;2016:5059041. doi: 10.1155/2016/5059041 (PMC4802027; doi:10.1155/2016/5059041)
Supplement: Supplementary file 1 — This file provides details regarding a PubMed search conducted on December 4, 2015 for citations indexed between the years 1990 and 2014 using the key term “meta-analysis”. Supplementary File 2: This file provides details regarding a PubMed search conducted on December 4, 2015 for citations indexed between the years 1990 and 2014 using the key terms “exercise and meta-analysis”. Supplementary File 3: This file is an example of the initial letter that was sent to authors requesting de-identified individual participant data for their previously published study. Supplementary File 4: This file is an example of the follow-up letter that was sent to eligible investigators describing what de-identified individual participant data was needed from their previously published study. [file 5059041.f1.pdf]

Supplementary File 1. PubMed searches using the keyword "meta-analysis".

Sign in to NCBI

PubMed.gov
US National Library of Medicine
National Institutes of Health

PubMed | meta-analysis | Search
Advanced
Help

Filters activated: Publication date from 1990/01/01 to 1990/12/31. Clear all

Search Details

Query Translation:

("meta-analysis"[Publication Type] OR "meta-analysis as topic"[MeSH Terms] OR "meta-analysis"[All Fields]) AND ("1990/01/01"[PDAT] : "1990/12/31"[PDAT])

Search URL

Result:

331

Translations:

meta-analysis	"meta-analysis"[Publication Type] OR "meta-analysis as topic"[MeSH Terms] OR "meta-analysis"[All Fields]

Database:

PubMed

User query:

meta-analysis AND (("1990/01/01"[PDat] : "1990/12/31"[PDat]))

You are here: NCBI > Literature > PubMed
Write to the Help Desk

GETTING STARTED
NCBI Education
NCBI Help Manual
NCBI Handbook
Training & Tutorials
Submit Data

RESOURCES
Chemicals & Bioassays
Data & Software
DNA & RNA
Domains & Structures
Genes & Expression
Genetics & Medicine
Genomes & Maps
Homology
Literature
Proteins
Sequence Analysis

POPULAR
PubMed
Bookshelf
PubMed Central
PubMed Health
BLAST
Nucleotide
Genome
SNP
Gene
Protein
PubChem

FEATURED
Genetic Testing Registry
PubMed Health
GenBank
Reference Sequences
Gene Expression Omnibus
Map Viewer
Human Genome
Mouse Genome
Influenza Virus
Primer-BLAST
Sequence Read Archive

NCBI INFORMATION
About NCBI
Research at NCBI
NCBI News
NCBI FTP Site
NCBI on Facebook
NCBI on Twitter
NCBI on YouTube

Sign in to NCBI

PubMed.gov
US National Library of Medicine
National Institutes of Health

PubMed | meta-analysis | Search
Advanced
Help

Filters activated: Publication date from 2014/01/01 to 2014/12/31. Clear all

Search Details

Query Translation:

("meta-analysis"[Publication Type] OR "meta-analysis as topic"[MeSH Terms] OR "meta-analysis"[All Fields]) AND ("2014/01/01"[PDAT] : "2014/12/31"[PDAT])

Search URL

Result:

15138

Translations:

meta-analysis	"meta-analysis"[Publication Type] OR "meta-analysis as topic"[MeSH Terms] OR "meta-analysis"[All Fields]

Database:

PubMed

User query:

meta-analysis AND (("2014/01/01"[PDat] : "2014/12/31"[PDat]))

You are here: NCBI > Literature > PubMed
Write to the Help Desk

GETTING STARTED
NCBI Education
NCBI Help Manual
NCBI Handbook
Training & Tutorials
Submit Data

RESOURCES
Chemicals & Bioassays
Data & Software
DNA & RNA
Domains & Structures
Genes & Expression
Genetics & Medicine
Genomes & Maps
Homology
Literature
Proteins
Sequence Analysis

POPULAR
PubMed
Bookshelf
PubMed Central
PubMed Health
BLAST
Nucleotide
Genome
SNP
Gene
Protein
PubChem

FEATURED
Genetic Testing Registry
PubMed Health
GenBank
Reference Sequences
Gene Expression Omnibus
Map Viewer
Human Genome
Mouse Genome
Influenza Virus
Primer-BLAST
Sequence Read Archive

NCBI INFORMATION
About NCBI
Research at NCBI
NCBI News
NCBI FTP Site
NCBI on Facebook
NCBI on Twitter
NCBI on YouTube

Supplementary File 2. PubMed searches using the keywords "exercise and meta-analysis".

Filters activated: Publication date from 1990/01/01 to 1990/12/31. Clear all

Search Details

Query Translation:

("exercise"[MeSH Terms] OR "exercise"[All Fields]) AND ("meta-analysis"[Publication Type] OR "meta-analysis as topic"[MeSH Terms] OR "meta-analysis"[All Fields]) AND ("1990/01/01"[PDAT] : "1990/12/31"[PDAT])

Search URL

Result:

7

Translations:

exercise	"exercise"[MeSH Terms] OR "exercise"[All Fields]
meta-analysis	"meta-analysis"[Publication Type] OR "meta-analysis as topic"[MeSH Terms] OR "meta-analysis"[All Fields]

Database:

PubMed

User query:

exercise AND meta-analysis AND (("1990/01/01"[PDat] : "1990/12/31"[PDat]))

Filters activated: Publication date from 2014/01/01 to 2014/12/31. Clear all

Search Details

Query Translation:

("exercise"[MeSH Terms] OR "exercise"[All Fields]) AND ("meta-analysis"[Publication Type] OR "meta-analysis as topic"[MeSH Terms] OR "meta-analysis"[All Fields]) AND ("2014/01/01"[PDAT] : "2014/12/31"[PDAT])

Search URL

Result:

472

Translations:

exercise	"exercise"[MeSH Terms] OR "exercise"[All Fields]
meta-analysis	"meta-analysis"[Publication Type] OR "meta-analysis as topic"[MeSH Terms] OR "meta-analysis"[All Fields]

Database:

PubMed

User query:

exercise AND meta-analysis AND (("2014/01/01"[PDat] : "2014/12/31"[PDat]))

Supplementary File 3. Initial email request to investigators.

Dear Dr. x:

We are currently funded by the National Institutes of Health (NIH), National Institute of Arthritis and Musculoskeletal and Skin Diseases (NIAMS) Grant #1-R01-AR061346, for the purpose of conducting an individual participant data (IPD) meta-analysis addressing the effects of exercise on depressive symptoms in adults with arthritis and other rheumatic diseases (AORD). I serve as the Principal Investigator on this project and am joined by Kristi Kelley (Research Technician), also from West Virginia University, and Dr. Jennifer Hootman (Co-Investigator) from the Centers for Disease Control and Prevention in Atlanta, Georgia. With the former in mind, you are listed as the corresponding author for the following nice study that meets our inclusion criteria:

Reference article here.

In this **preliminary** correspondence, we would like to know if you would be willing to participate in our study by sharing your IPD with us, void of any personal identifying information such as name, social security number, etc. In order to reduce your burden, we would ask that you supply this IPD in whatever format is most convenient for you (Excel, SPSS, SAS, Stata, text, etc.). In addition, we would also need a description of all coded variables that were provided to us. You also have the option of simply supplying us with the entire dataset if that would be easier for you.

Our Office of Sponsored Projects' Institutional Review Board has deemed this project as not human subject's research.

If you agree to supply us with your IPD, and we most certainly hope that you will, we will send a follow-up E-mail with specifics. In agreeing to supply us with your IPD, we would acknowledge your willingness to do so by including your name in the Acknowledgements section of any manuscripts and presentations in which your work is included as well as our required reports to NIH.

We see this research as an opportunity to better understand and quantify the effects of exercise on depressive symptoms in adults with AORD as well as to provide direction for future researchers such as you.

If you could please respond to this E-mail within **two weeks**, we would greatly appreciate it. Also, if you have any questions, please feel free to contact me directly at any time.

Take care and we look forward to hearing of your willingness to participate.

Sincerely yours,

George (Kelley)

George A. Kelley, DA, FACSM
Professor & Director, Meta-Analytic Research Group
School of Public Health
Department of Biostatistics
PO Box 9190
Robert C. Byrd Health Sciences Center
Room 2350-A
West Virginia University
Morgantown, WV 26506-9190
Office Phone: 304-293-6279
Lab Phone: 304-293-6280
Fax: 304-293-5891
E-mail: gkelley@hsc.wvu.edu
Website: http://publichealth.hsc.wvu.edu/pages/Personnel/Faculty

Supplementary file 4. Data request for individual participant data.

THANK YOU SO MUCH for agreeing to supply us with your de-identified individual participant data (IPD) for our meta-analytic research addressing the effects of exercise on depressive symptoms in adults with arthritis and other rheumatic diseases (AORD), funded by the National Institutes of Health (NIH), National Institute of Arthritis and Musculoskeletal and Skin Diseases (NIAMS) Grant #1-R01-AR061346. With the former in mind, we are requesting the information listed below, including definitions and descriptions of all variables provided to us, preferably in the English language. In order to ease the burden on you, we will accept your information in whatever format you prefer (Excel, SPSS, SAS, Stata, text, etc.). **When providing data, please make sure that there is a differentiation between all time points assessed (for example, pre and post) as well as group assignment (for example, intervention and control).** If information for some of the variables requested below is not available or was not measured, please supply us with the information you have for the other variables. Please note that you also have the option of simply supplying us with the entire dataset if that would be easier for you. Here are the data that we are requesting at the level of the individual:

A. Participant characteristics
1. Age
2. Gender
3. Race/ethnicity
4. Hyperlipidemic (yes or no)
5. Prehypertensive (yes or no)
6. Hypertensive (yes or no)
7. Type 1 diabetes (yes or no)
8. Type 2 diabetes (yes or no)
9. Cardiovascular disease (yes or no)
10. Stroke (yes or no)
11. Metabolic syndrome (yes or no)
12. Asthma (yes or no)
13. Rheumatoid arthritis (yes or no)
14. Number of years since diagnosis of arthritis
15. Number of years since symptoms of arthritis appeared
16. Osteoarthritis (yes or no)
17. Fibromyalgia (yes or no)
18. Osteopenia (yes or no)
19. Osteoporosis (yes or no)
20. Menopausal status (pre, post, not applicable)
21. Cigarette smoking (yes or no)
22. Alcohol consumption (yes or no)
23. Medications taken (list any medications taken)
24. Marital status (as you've defined)
25. Educational attainment (as you've defined)

B. Exercise program (aerobic)
1. Length (total weeks of training)
2. Frequency (number of sessions attended per week)
3. Intensity (%VO_{2max}, %maximum heart rate, % maximum heart rate reserve, METS, RPE, etc.)
4. Duration (minutes of exercise per session)
5. Mode(s) of training (for example, jogging)
6. Number of steps (if pedometer used)
7. Compliance to the exercise intervention (percentage of exercise sessions attended)
8. Injuries incurred as a result of the aerobic exercise intervention
9. Adverse events incurred as a result of the aerobic exercise intervention

C. Exercise program (resistance training)
1. Length (weeks)
2. Frequency (number of sessions attended per week)
3. Intensity (for example, %1RM)
4. Number of sets per exercise
5. Number of repetitions per exercise
6. Rest between sets (reported as seconds or minutes)
7. Number of exercises performed
8. List of exercises performed (for example, bench press, arm curls, etc.)
9. Compliance to the exercise intervention (percentage of exercise sessions attended)
10. Injuries incurred as a result of the resistance training intervention
11. Adverse events incurred as a result of the resistance training intervention

D. Outcomes
1. Depressive symptoms score (primary)
2. Physical function (secondary)
3. Pain (secondary)
4. Anxiety (secondary)
5. Self-worth/self esteem
6. Quality of life
7. Height (other)
8. Body weight (other)
9. Body mass index (BMI) (other)
10. Percent body fat (other)
11. Fat mass (other)
12. Lean body mass (other)
13. Energy intake (other)
14. Energy expenditure (other)
15. Physical activity level, excluding the intervention (other)
16. Aerobic fitness (for example, maximum oxygen consumption) (other)
17. Muscular strength (other)
18. Muscular endurance (other)
19. Muscular power (other)
20. Static balance (other)
21. Dynamic balance (other)
22. Flexibility (other)

Please E-mail all data within **4 weeks** to gkelley@hsc.wvu.edu Again, **THANK YOU.**
